# ATP transporters in the joints

**DOI:** 10.1007/s11302-021-09810-w

**Published:** 2021-08-15

**Authors:** Ane Larrañaga-Vera, Miguel Marco-Bonilla, Raquel Largo, Gabriel Herrero-Beaumont, Aránzazu Mediero, Bruce Cronstein

**Affiliations:** 1 Department of Medicine, Division of Translational Medicine, NYU Langone Health, New York, NY USA; 2 Bone and Joint Research Unit, IIS-Fundación Jiménez Díaz UAM, 28040 Madrid, Spain

**Keywords:** Cx43, Panx1, Panx3, Joint diseases

## Abstract

Extracellular adenosine triphosphate (ATP) plays a central role in a wide variety of joint diseases. ATP is generated intracellularly, and the concentration of the extracellular ATP pool is determined by the regulation of its transport out of the cell. A variety of ATP transporters have been described, with connexins and pannexins the most commonly cited. Both form intercellular channels, known as gap junctions, that facilitate the transport of various small molecules between cells and mediate cell–cell communication. Connexins and pannexins also form pores, or hemichannels, that are permeable to certain molecules, including ATP. All joint tissues express one or more connexins and pannexins, and their expression is altered in some pathological conditions, such as osteoarthritis (OA) and rheumatoid arthritis (RA), indicating that they may be involved in the onset and progression of these pathologies. The aging of the global population, along with increases in the prevalence of obesity and metabolic dysfunction, is associated with a rising frequency of joint diseases along with the increased costs and burden of related illness. The modulation of connexins and pannexins represents an attractive therapeutic target in joint disease, but their complex regulation, their combination of gap-junction-dependent and -independent functions, and their interplay between gap junction and hemichannel formation are not yet fully elucidated. In this review, we try to shed light on the regulation of these proteins and their roles in ATP transport to the extracellular space in the context of joint disease, and specifically OA and RA.

## Joint diseases

A number of metabolic, immunologic, inflammatory, and degenerative diseases affect the structures of the joint [1]. They result in reduced mobility and can be extremely painful and debilitating as they progress toward mobility failure. These problems are a leading cause of disability worldwide, representing a principal cause of dependence in the elderly, and can lead to increased mortality [2, 3]. Two of the most common disorders of the joint are osteoarthritis (OA) and rheumatoid arthritis (RA) [4, 5]. The socioeconomic burden of these pathologies is enormous; by 2040, nearly 78 million US adults are expected to have some form of arthritis diagnosed [6]. OA, the most common form of arthritis, affected up to 14% US adults (32.5 million) in 2008–2014, according to United States Bone and Joint Initiative [7], and was responsible for 2.4% of all healthcare visits in that period. RA, an autoimmune form of inflammatory arthritis, has an estimated prevalence of 2% among the US population 60 years of age and older [8]. OA is predominantly a degenerative disease that can affect almost any joint, but generally affects the hands, knees, hips, and feet, aging is its primary risk factor [9]. OA causes cartilage breakdown, subchondral bone sclerosis, osteophyte formation, and synovial inflammation, leading to joint pain and physical disability in the aging population [10]. RA is a chronic autoimmune disease that can affect any joint, but generally involves the large and small joints of the hands, wrists, and feet and tends to be symmetrical in its effects [11]. RA is characterized by pain, swelling, and destruction of bones and cartilage, ultimately leading to joint destruction and disability [12]. It is primarily driven by a significant synovial inflammatory response involving the innate and adaptive immune system [13], in which fibroblasts in the synovium transform to an invasive phenotype, and inflammatory cells induce cartilage destruction and osteoclast generation that result in bone erosion, a hallmark of RA [14]. Although the two diseases differ in etiology, both trigger progressive joint destruction characterized by pathological changes in articular cartilage, bone, and synovium [5].

Articular cartilage is responsible for reducing friction and cushioning the impact produced during movement [15]. This avascular, aneural, and alymphatic tissue provides an elastic and resistant structure on the surface of the joints. Cartilage is formed by a single cell type, the chondrocyte, which secretes the matrix [16].

Underlying bone, separated from the cartilage by the cement line, is constituted of a thin cortical lamella and the underlying trabecular cortical bone [17]. Bone is a mineralized connective tissue comprising three main types of cells: osteoblasts, osteocytes, and osteoclasts. Bone exists in a constant state of remodeling, which is important for the maintenance of normal skeletal structure and function. Osteoblasts synthesize the matrix and play a major role in bone mineralization, formation, and deposition; osteoclasts are multinucleated giant cells that originate from the hematopoietic lineage and are responsible for the resorption of the mineralized bone matrix [18]. Osteocytes are derived from osteoblasts, are embedded within the mineralized matrix and are connected to each other by an intricate network of canalicular channels that coordinate the actions of osteoblasts and osteoclasts via the production of signaling molecules and sense mechanical stress [19].

The synovial membrane is the soft tissue lining the cavities of diarthrodial joints. Its main function is to produce the synovial fluid that minimizes joint wear and provides nutrients to the cartilage. In joint disease, the aggressive transformation of synoviocytes produces an inflammatory infiltrate that can destroy articular cartilage and cause bone erosion [14].

As the world population ages, the incidence of joint diseases is projected to rise significantly in future [7]. Therefore, increasing efforts are underway to find new treatments. There has been significant success in treating RA with drug therapy, including the introduction of new biologic and small-molecule drugs. In contrast, no new medical therapies for OA have been developed, although advances in orthopedic surgery can offer symptomatic relief for many patients.

## ATP transporters

Extracellular adenosine triphosphate (ATP), a leading extracellular signaling molecule acting in diverse physiological and pathological processes, has been shown to have a role in joint disease. Adenine nucleotides act as autocrine and paracrine signaling molecules at a large number of purinergic receptors (P2 receptors) that are expressed to varying degrees on different cell types. Extracellular nucleotidases regulate extracellular ATP levels by dephosphorylating ATP to adenosine, which acts as an agonist at P1 receptors (a second class of purinergic receptors) [20].

As in other systems, released ATP in the bone microenvironment binds to purinergic receptors in an autocrine or paracrine manner. In the immune system, ATP released from damaged cells promotes cell migration through paracrine signaling [21, 22], whereas ATP released from migrating cells enhances cell motility in an autocrine manner [22–24]. This autocrine loop is essential for macrophage chemotaxis [25]. At least nine possible autocrine loops exist in resident peritoneal macrophages, with any one of three redundant feedback loops terminating on P2Y2, P2Y12, or adenosine receptors sufficing for efficient chemotaxis [25, 26]. In the absence of pannexin-1 (Panx1), P2Y2 or P2Y12 mice exhibited efficient chemotactic navigation. This is blocked in the presence of apyrase as it degrades ATP and ADP, and also with the inhibition of multiple purinergic receptors [25]. This chemotactic function of the purinergic loop is activated in conditions such as inflammation, infection or cell necrosis [24]. Another example can be observed in microglia, where cells release ATP as positive feedback to the increased extracellular ATP, via lysosomes. This local signal can be amplified to induce migration of remote microglia, contributing to regenerative ATP signaling in the brain [24]. Chemoattraction also occurs in dendritic cells, where the activation of the P2X7 receptor by ATP, through Panx1 induces plasma membrane permeabilization contributing to the release of ATP [27]. This autocrine purinergic receptor signaling is also essential to amplify the action of platelet activators [28, 29].

Several different mechanisms exist to transport ATP out of the cell to the extracellular space, including channel-dependent mechanisms such as those involving connexin (Cx) hemichannels and pannexin (Panx) channels [30, 31].

### Connexins

Connexins are composed of four transmembrane domains, two extracellular loops, and intracellular N- and C-termini. Connexins are a family of proteins that form hemichannels (or connexons) that can in turn form intercellular gap junctions. More than 20 human connexin isoforms with differential functional properties and tissue distribution have been identified. They are commonly denoted by the word connexin, abbreviated Cx, followed by the molecular weight [32]. Connexin hemichannels are formed by the oligomerization of six connexins, which can be six monomers of the same connexin or a combination of different connexins [33]. Two opposing hemichannels on adjacent cells can form a gap junction that facilitates heterocellular communication [31].

Gap junctions and their importance in cell-to-cell transport and communication have been extensively studied [34]. The functionality of unopposed hemichannels, although a newer concept, is also well documented. In brief, connexin hemichannels form pores in the cell membrane that allow the transport of small molecules such as ATP, amino acids, reduced glutathione, NAD^+^, IP_3_, prostaglandin E_2_, and cyclic nucleotides to the extracellular space [35]. They can switch between open or closed conformation, and their pore, which is closed by default, can be induced to open in response to various stimuli, including changes in extracellular calcium concentrations, cytosolic pH, or stress conditions [33, 36].

Several connexin isoforms have been reported to be capable of releasing ATP through unopposed hemichannels from different cell types, including Cx26, Cx30, Cx32, Cx36, Cx40, and Cx43, with Cx43 being the best studied [30, 37–39].

### Pannexins

Pannexins are the human homologues of innexins, the proteins that constitute invertebrate gap junctions. Three isoforms, Panx1, Panx2, and Panx3, have been identified, although Panx1 is the most ubiquitously expressed and the best studied. Pannexin channels are involved in releasing extracellular ATP as a paracrine signaling ligand associated with inflammatory events [40, 41]. Panx1 can be activated and release ATP at physiological Ca^2+ ^concentrations and membrane potentials in response to mechanical stimulation, caspase cleavage, or extracellular K^+^ [42–45].

Pannexins exist in the membrane as tetraspan proteins with intracellular N- and C-termini. Panx1 can be found as a hexamer and was recently described as an heptamer [46–48], while Panx3 forms hexameric single-membrane channels, and Panx2 is predicted to exist as heptamers and octomers. Pannexins can function as single-membrane channels and do not form gap junction channels [49, 50].

Shestopalov and Panchin have reported that Panx1 is expressed broadly, whereas Panx2 is expressed mainly in brain, and Panx3 in skin and connective tissues [51]. Northern blot studies indicate that Panx1 is ubiquitously expressed in human tissues, including the brain, lung, liver, skin, heart, skeletal muscle, spleen, thymus, pancreas, and colon [52]. Using custom-designed anti-Panx1 antibodies, Panx1 expression has been found in the human brain, with variable levels in the lung, spleen, kidney, heart ventricle, and skin and in murine ear and tail cartilage [53]. Based on expressed sequence tags, mammalian Panx3 has been identified in synovial fibroblasts, osteoblasts, joints in murine paws, inner ear cartilage, and cochlear bone [52, 54]. Moreover, Panx3 has been detected by in situ hybridization in pre-hypertrophic chondrocytes, perichondrium and osteoblasts of embryonic day (E) 16.5 mice [55]. All three pannexin subtypes have been identified in several cultured mouse cell lines, including osteoblast (MC3T3-E1), chondrocyte (ATDC5), and osteoprogenitor cell lines (C2C12), as well as in primary osteoblasts [56] (Table 1).

**Table 1:** Roles of connexin and pannexin channels in cartilage and bone cell lines

Channel	Cell type	Function
Cx43	Chondrocytes	• OA chondrocytes have increased Cx43 expression compared to healthy chondrocytes [63] • The inflammatory environment in rheumatic joints enhances Cx43 function in cartilage [38]
	Synoviocytes	• OA synoviocytes have increased Cx43 expression compared to healthy synoviocytes [63] • Cx43 induces the expression of OA-associated genes such as *MMP* genes or *ADAMTS* [81]
	Osteoblast	• Cx43 inhibits osteoblast precursor proliferation [89, 90] • Cx43 is necessary for the anabolic proprieties of PTH [92–94] • Diminished Cx43 levels influence the activity of bisphosphonates, reducing their anti-apoptotic effects on osteoblasts [95]
	Osteocytes	• Cx43 hemichannels are essential for osteocyte viability [86] • Cx43 is responsible for the mechanosensing properties of osteocytes by promoting ATP release [97]
	Osteoclasts	• Cx43 is central to cell fusion in osteoclastogenesis in vitro [101]
Panx1	Chondrocytes	• Panx1 mediates cell-to-cell interaction in response to cell stiffness [63]
	Osteocytes	• Panx1 forms a complex with the P2X7 receptor that promotes NLRP3 inflammasome activation [110] • Panx1 enhances RANKL expression under apoptotic conditions [112] • Panx1 enhances bone resorption in response to apoptosis [116]
	Osteoclast	• Panx1 is essential for osteoclast differentiation [114]
	Osteoblast	• Panx1 increases RANKL expression [112]
Panx2	Osteoblast	• Panx2 expression levels do not change during osteoblast differentiation [132]
Panx3	Chondrocytes	• Panx3 promotes chondrocyte differentiation by regulating intracellular ATP/cAMP levels [55] • Panx3 inhibits cell proliferation [55] • Panx3 induces ATP release during joint damage and triggers cartilage and joint destruction in OA [136–138]
	Osteoblast	• Panx3 promotes osteoblast differentiation [134] • Panx3 is not required for postnatal bone remodeling [132]

Panx1 can be inactivated by ATP release, CO_2_-mediated cytoplasmic acidification, channel blockers, or mimetic peptides [57, 58]. Another regulatory mechanism that has been proposed is phosphorylation of Panx1. During acute vascular inflammation, Panx1 phosphorylation at tyrosine 198 by Src-family tyrosine kinases results in increased channel activity [59]. Sustained neuronal depolarization is mediated by Panx1 activation thought phosphorylation of tyrosine 308, allowing the activation of NMDA receptors during anoxia [60]. Panx1 phosphorylation can also mediate decreases in channel activity, as observed in HEK-293 human embryonic kidney cells in which Panx1 channels were inhibited by nitric oxide [61].

Although there is considerable information available on the actions of connexins and pannexins in cellular ATP release, determining their relative contributions to ATP release and other signaling events is a major challenge in the field [40, 41]. In this review, we focus on the expression and activity of these channels in joint diseases.

## ATP transporters in joint diseases

### Cx43 in the joint

Although diverse connexins have been identified in joint tissues, Cx43 is the most widely expressed, found in chondrocytes, synovial fibroblasts, and bone cells, and it is considered to play a role in a variety of musculoskeletal pathologies including OA, RA, and osteoporosis.

**Fig. 1:**
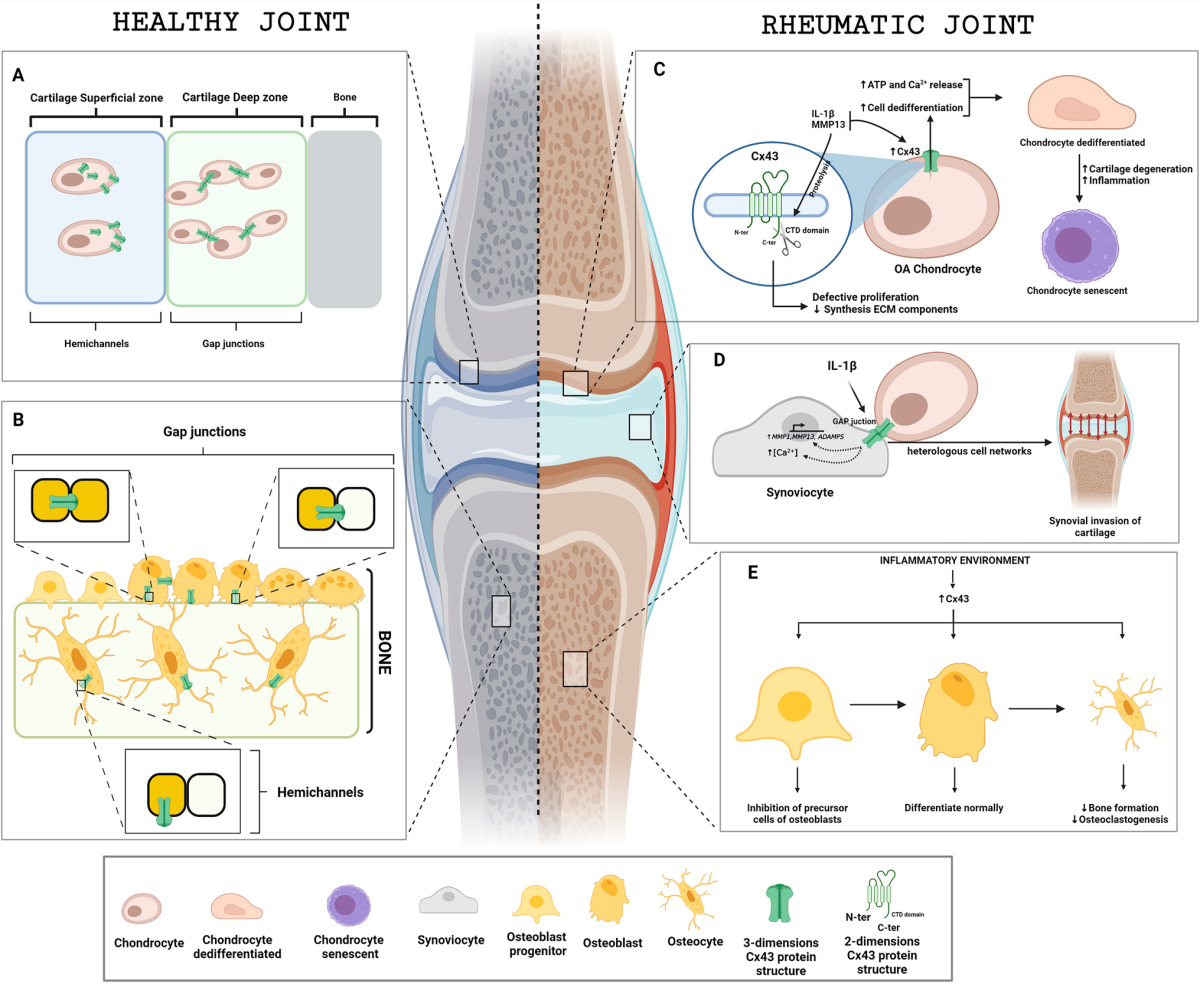
Role of connexin 43 (Cx43) in healthy and arthritic joints. A) Under homeostatic conditions, the distribution of Cx43 (green structures) in cartilage depends on the location of chondrocytes in the tissue. In the area near the synovial capsule, Cx43 has formed hemichannels in chondrocytes. In contrast, the deep zone of the cartilage contains more abundant chondrocytes that allow the formation of gap junctions, favoring cell-to-cell interaction. B) In bone, Cx43 in osteoblasts forms both hemichannels and gap junctions to interact with proximal osteoblasts. In osteocytes, Cx43 alone maintains the formation of hemichannels. C) The inflammatory environment of an arthritic joint favors the expression of MMP13 and IL-1β. This increase is related to a rise in Cx43 in chondrocytes, which leads in to the release of calcium and ATP and to a dedifferentiation process that promotes chondrocyte senescence due to progressive degradation of the cartilage. D) The increase in Cx43 due to increased IL-1β allows the overexpression of genes associated with OA, such as *MMP1*, *MMP13*, and *ADAMPS*, and the release of intracellular calcium. It also promotes interaction with proximal chondrocytes through the formation of gap junctions, which leads to the proliferation of synoviocytes in the cartilage. E) In bone, inflammatory conditions together with higher Cx43 abundance lead to the inhibition of osteoblast precursors and a decrease in bone formation and osteoclastogenesis in osteocytes in vitro

One of the most important hallmarks of OA and RA is the destruction of cartilage, and Cx43 is broadly expressed in chondrocytes, which produce and maintain cartilage. Because these cells are commonly found in individual lacunae, Cx43 is more likely to be forming hemichannels that do not constitute gap junctions; indeed, the presence of hemichannels in chondrocytes has been confirmed in vitro (Fig. 1) [62]. However, the specific role of connexins in particular pathologies remain to be fully elucidated.

Chondrocytes from patients with OA are reported to express more Cx43 than healthy chondrocytes [63]. This phenomenon might be a consequence of the activation of a repair response, also observed in wound healing, that may maintain chondrocytes in a more immature, proliferative state. Although increased Cx43 expression enables constant ECM remodeling, in the long term increased Cx43 expression and function could lead to the gradual formation of poor-quality fibrocartilage (Fig. 1) [64].

Connexin hemichannels are reported to respond to cyclical mechanical loading in cartilage by increasing the concentration of ATP in the extracellular space, which would activate P2 receptors, calcium signaling cascades and, after ATP is dephosphorylated to adenosine, P1 receptors. This increase in ATP release is significantly reduced in the presence of flufenamic acid, an hemichannel inhibitor [65].

Cx43 hemichannels function as mechanosensitive ATP-release channels in different cell types [66–68]. Both bovine and human explant chondrocytes express Cx43 in the superficial region, down to approximately 200 μm below the articular surface [62]. This suggests that Cx43 performs uncoupled mechanotransduction primarily in the superficial/middle zone of articular cartilage through mechanotransduction pathways different from those operating in the cells in deeper zones. Moreover, this could be related to the way that (as shown in other cell types) hypoxia regulates Cx43 dephosphorylation, translocation, and proteosomal degradation [69]. Suggestively, the size of this subpopulation of mechanosensitive chondrocytes expressing Cx43 in the cilium (~ 15%) is very similar to that of the subpopulation showing mechanosensitive purinergic Ca^2+^ signaling [70]. ATP may activate P2 receptors and thereby activate intracellular Ca^2+^ signaling cascades that have an anabolic effect, upregulating proteoglycan and collagen synthesis and cell proliferation [71, 72]. In contrast, chondrocytes isolated from OA cartilage do not show mechanically induced ATP-mediated hyperpolarization, even though P2Y2 mRNA expression is similar in normal and OA cells [72]. This indicates that the abnormalities of mechanotransduction in OA are not related to differences in P2 receptor expression but rather involve ATP desensitization due to an increased levels of ATP in the synovial fluid in OA [72, 73]. Moreover, Cx43 has been detected in meniscal cell clusters, suggesting that it may be connected to the development of OA [74, 75]. Furthermore, Cx43 and Cx45 levels within the damaged superficial zone and middle zone cartilage are elevated in patients with OA [62].

Cx43 might also be acting through channel-independent mechanisms in OA, working as a scaffold protein [76] binding to cytoskeletal proteins and regulating cytoskeletal architecture and cell proliferation [77, 78].

The inflammatory environment in rheumatic joints can further contribute to the regulation of Cx43 in cartilage. Inflammation, similarly to mechanical load, causes increased production and release of ATP by opening plasma membrane hemichannels; this leads to the activation of purinoceptors, resulting in increased calcium release [79]. Interleukin 1 (IL-1) is the best-studied cytokine associated with rheumatic diseases and is well established to promote cartilage destruction. In rabbit articular chondrocytes, IL‐1 increases cytosolic Ca^2+^ concentrations, resulting in overexpression of Cx43. Tonon et al. have suggested that IL-1 could increase the abundance of gap junctions between chondrocytes and synoviocytes, forming heterologous cell networks and favoring synovial invasion of the cartilage (Fig. 1) [80]. However, these experiments were performed under mechanical stimulation, and the potential role of hemichannels cannot be fully established without further studies.

Another proposed role of Cx43 on chondrocytes relates to the hemichannel C-terminal domain (CTD). Recent studies reveal that CTD deficiency in mice alters chondrocyte structure and phenotype, leading to defective cellular proliferation and decreased synthesis of matrix components. Cx43 is a substrate for a variety of metalloproteases (MMP) that are upregulated in the joints of individuals with OA as a consequence of inflammation. MMP proteolysis of CTD may contribute to the phenotypic changes associated with functional alterations of Cx43 that lead to defective tissue repair and disease progression, especially considering that CTD is a pivotal player in regulating the chemical gating of Cx43 channels that influence the release of ATP [78].

Similar to cartilage, OA synovial membrane also shows increased Cx43 expression as compared to healthy tissue. In synoviocytes, Cx43 induces the expression of OA-associated proteins such as MMP-1, MMP-13, and ADAMTS, a disintegrin-like and metalloproteinase with a thrombospondin type 1 motif (Fig. 1) [81].

Moreover, a collagen-induced arthritis rat model of RA [79] showed increased synovial Cx43 expression compared to control rats. In vitro lipopolysaccharide inflammatory stimulus also enhanced Cx43 expression, and the MMP increase in response to IL-1 was shown to be Cx43 dependent in cultured synovial fibroblasts [82, 83]. More importantly, in a genetic silencing study of Cx43, rats treated with short interfering RNA directed against the *Cx43* gene (siCx43) showed reductions in joint swelling, arthritis scores, and numbers of osteoclast‐like cells. In culture, siCx43 reversed the lipopolysaccharide (LPS)-induced upregulation of TNF‐α, IL‐6, and IL‐1β mRNAs [83].

Human patients with oculodentodigital dysplasia (ODDD) have mutations in Cx43 and show craniofacial abnormalities, aplastic or hypoplastic middle phalanges, syndactyly, and broad tubular long bones, indicating that connexins have a role in skeletal development [33]. Esseltine et al. obtained induced pluripotent stem cells (iPSCs) from a patient with a connexin‐linked ODDD and found that, compared to wild-type iPSCs, they showed reduced Cx43 mRNA and protein and impaired channel function, which translated to delayed osteoblast differentiation, along with delayed expression of collagen‐I bone sialoprotein (BSP) and osteopontin (OPN) in differentiated ODDD cells [84]. However, some mutations found in patients with ODDD can lead to reductions in gap junctions but also a hemichannel gain of function [85].

To discriminate between the roles of hemichannels and gap junctions in ODDD, two mouse models expressing dominant negative mutants of Cx43 have been developed. The first, R67W, has the ability to form functional hemichannels, but not gap junction channels, whereas the second, Δ130–136, has complete loss of Cx43 function [86]. The researchers found that while Δ130–136 mice showed reduced bone mass and aberrant bone structure, R67W mice had only a few abnormalities. However, they did not evaluate the gain or loss of hemichannel functionality.

Other mouse models have also been developed to help elucidate the function of Cx43 in bone. Mice with total Cx43 knockout (KO) die as a consequence of a cardiac phenotype, but during embryonic development, their bones show reduced mineralization, cortical thinning, and increased porosity [87, 88]. Meanwhile, studies using conditional KO models have pointed to differential roles of Cx43 at different stages of osteoblastic maturation. It has been suggested that Cx43 might inhibit the proliferation of early osteoblast precursor cells, as conditional Cx43KO mice show increases in this populations. However, when Cx43 is knocked out in lineage-committed cells, osteoblasts do not seem to be essential and cells differentiate normally [89, 90]. Cx43-deficient mice also show impaired osteoblast differentiation and fracture repair (Fig. 1) [91].

Age-associated reduction of Cx43 in osteoblasts has been linked to bone loss and osteoporosis, and it could diminish parathyroid hormone (PTH)-dependent bone formation. Moreover, Cx43 seems to be essential for the anabolic proprieties of PTH when used to treat osteoporosis, as it prevents PTH-induced production of cAMP by osteoblastic cells [92–94].

Diminished Cx43 levels in osteoblast and osteocytes could also influence the activity of bisphosphonates, the most commonly prescribe anti-osteoporotic drugs, by reducing their anti-apoptotic effects on these cells [95]. Bisphosphonates are reported to trigger the activation of the kinases Src and ERKs, which promotes cell survival by opening Cx43 hemichannels in a gap-junction-independent manner [96]. Moreover, bisphosphonate treatments that cause increased bone mineral density induce the formation of Cx43 hemichannels in osteocytes, which can result in increased ATP release.

Cx43 is considered essential for osteocyte viability and bone health, based on evidence from Δ130–136 and R67W mice, which also suggested that this effect is mediated by hemichannels rather than gap junctions [86]. Cx43 is also considered central to mechanosensing capability of osteocytes. Osteocytic MLO-Y4 cells express functional hemichannels that are activated by oscillating fluid flow through a mechanism that involves protein kinase C and promotes ATP release [97].

Conversely, although in vitro studies showed that Cx43-deficient cells have reduced responsiveness to biomechanical signals, Cx43-deficient mice display an enhanced anabolic response to mechanical load in vivo [98, 99]. This could be explained by a mechanism in which Cx43 deficiency leads to enhanced bone formation and resorption as well as enhanced response to load and decreased response [100].

In line with this, Zhang et al. reported that mice with osteocyte-specific Cx43 deficiency showed increased bone resorption and osteoclastogenesis due to osteocyte-mediated changes in RANKL/OPG ratio [99]. Further, Cx43 is believed to influence osteoclastogenesis, allowing cell fusion, as blocking gap-junctional communication inhibits bone resorption in vitro [101]. However, no direct function of hemichannels in osteoclastogenesis has been described, and in vivo studies will be required to demonstrate whether they play a role.

### Panx1 in the joint

**Fig. 2:**
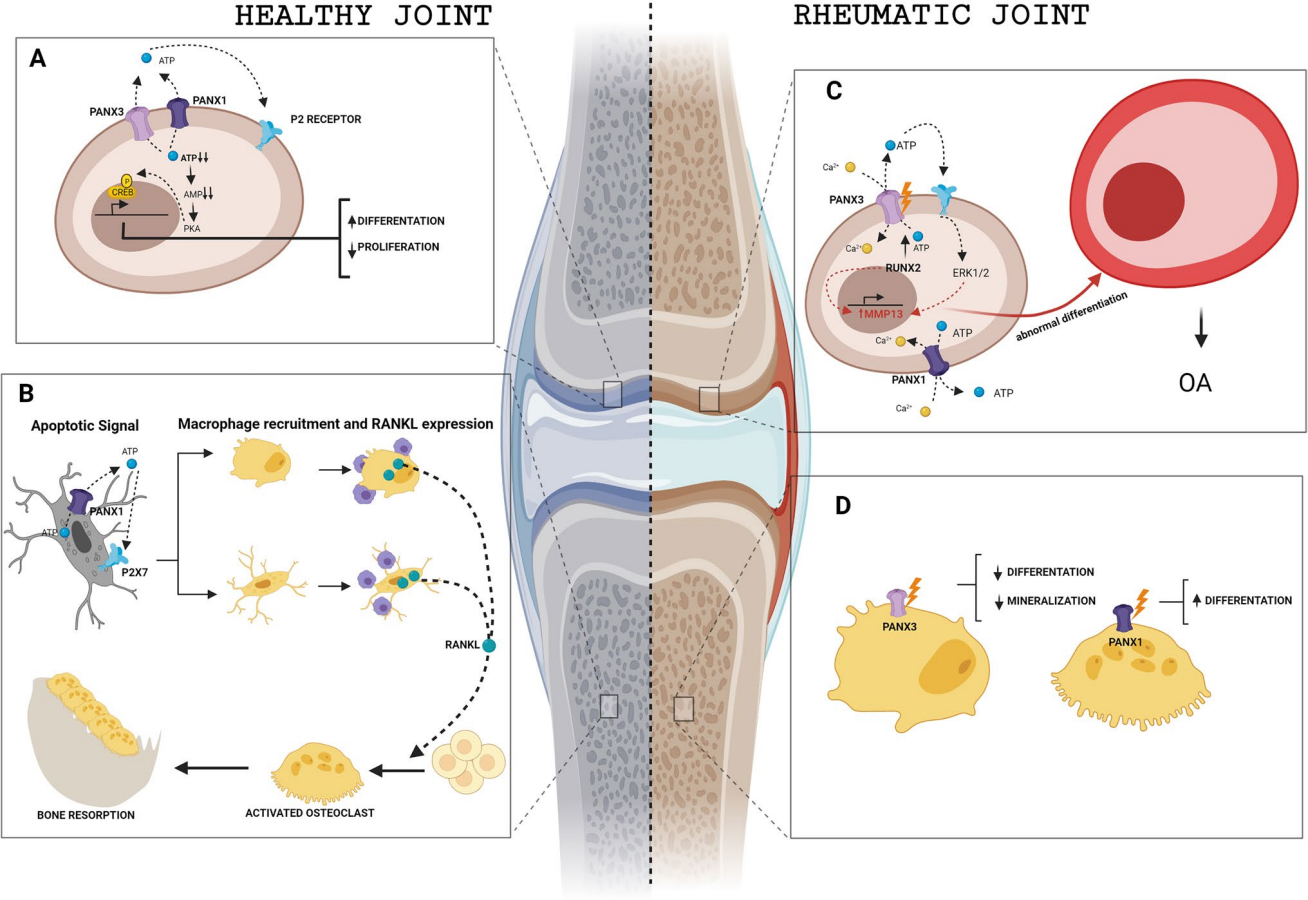
Role of pannexin channels in healthy and arthritic joints. A) In healthy cartilage, chondrocytes mediate ATP release by Panx3. This leads a decrease in intracellular ATP, activation of phosphokinase A, and phosphorylation of CREB, a transcription factor that induces cell proliferation inhibition. B) In bone, the release of ATP by Panx1 is a signal of osteocyte apoptosis, causing macrophage recruitment and RANKL release that enables osteoclast activation and increased bone resorption. C) In rheumatic disease, the release of ATP mediated by Panx3 via Runx2 in chondrocytes activates P2 receptors. This leads to a repetitive cascade of signaling mediated by ERK1/2 and MMP13, which direct an aberrant differentiation to hypertrophic chondrocyte phenotype. D) Rheumatic bone disease alters Panx3 activity in osteoblasts and Panx1 activity in osteoclasts

All three pannexins have been detected in murine chondrocytes, with Panx1 most abundantly expressed (Fig. 2). Studies using polydimethylsiloxane of varied stiffness as a cell culture substrate indicate that cell–cell communication in chondrocytes is mediated by Panx1 in response to stiffness and that this is critical for chondrocyte metabolism and cartilage tissue engineering [63, 102].

Joint pain during arthritis is produced by aberrant nociceptive circuits activated in the central nervous system [103], and ATP is released during tissue injury and critically modulates microglial activity [104]. Mousseau et al. found that joint injury is followed by an increase in nociceptive output that depends on Panx1 expressed on microglia. They observed that pharmacological and genetic inhibition of Panx1 ameliorated mechanical joint pain in rodents. Moreover, continuous systemic treatment with probenecid (a US FDA–approved broad-spectrum Panx1 inhibitor) prevented the reduction in mechanical threshold that occurs in rats with monosodium iodoacetate (MIA)-induced joint injury (which mimics arthritis) and blocked the increase in IL-1β that plays a dominant role in promoting joint pain [105].

The functional role of Panx1 channels is cell type specific. Extracellular ATP is sensed by P2X7 and P2X4 receptors and mediates activation of the NLRP3 inflammasome during inflammation [106–108]. The NLRP3 inflammasome is a multiprotein complex that takes part in the innate immunity machinery [109] and whose activation leads to activation of intracellular caspase 1 and secretion of pro-inflammatory IL-1β and IL-18. Panx1 directly interacts with the P2X7 receptor, and this interaction has been associated with NLRP3 activation [110, 111]. The P2X7/Panx1 complex directly binds to caspase-1 and induces IL-1β release and pyroptosis in neurons and astrocytes [108]. Pharmacological and genetic inhibition of the P2X7 receptor ameliorates mechanical joint pain in rodents in a similar manner to the blockade of Panx1 [105].

Moreover, the caspase-mediated activation of these pathways by the Panx1/P2X7 receptor complex and ATP release serves as a ‘find me’ signal necessary for macrophage recruitment during apoptosis [42]. Panx1 activation and ATP release in apoptotic osteocytes, activated by the P2X7 receptor, enhances RANKL expression in neighboring osteocytes and osteoblasts, induces macrophage recruitment, and increases osteoclast abundance in adjacent bone surface [112]. This cell fusion during osteoclast maturation is due to P2X7/Panx1-mediated ATP release that also enhances bone resorption [113–115]. The attenuation of Panx1 bone resorption that occurs in Panx1 KO mice after osteocyte apoptosis indicates that Panx1-mediated ATP release is a prerequisite for bone resorption [116].

Osteocyte apoptosis triggers bone remodeling by inducing neighboring viable osteocytes to produce RANKL [117] (Fig. 2). This process begins early in apoptosis, suggesting that signals involved in apoptosis play a key role in inducing viable osteocytes to produce osteoclastogenic cytokines. ATP and UTP together with lysophosphatidylchloline (lysoPC) and the chemokine CX3CL1 have been identified as ‘find me’ signals in the early phase of apoptosis [118]. Osteocytes co-express Panx1 and the P2X7 receptor and activate caspase-dependent Panx1 activation and channel opening during apoptosis [97, 119]. Work in Panx1 KO animals has demonstrated that Panx1 does not participate in osteocyte apoptosis mediated by bone fatigue and microdamage, but is essential for RANKL expression triggered by apoptotic osteocytes and the initiation of osteoclastic bone remodeling. Similarly, work in P2X7 receptor knockdown mice indicated that both the P2X7 receptor and Panx1 are essential during osteocyte apoptosis and RANKL release associated with fatigue and microdamage [116]. Although the exact mechanism involved is not clear, it is likely to be a paracrine mechanism whereby ATP is released via caspase-3-mediated Panx1 opening in apoptotic osteocytes and activating the P2X7 receptor in viable osteocytes [116]. Because Panx1 and P2X7 receptor KO are total in the mouse system used, and both genes are ubiquitously expressed, alterations in other cells may contribute to RANKL expression and bone remodeling in microdamage [56]. This finding is consistent with results indicating that both Panx1 and the P2X7 receptor are essential for osteoclast differentiation and macrophage fusion in vitro [114].

During bone digestion, membrane components of the ruffled border also need to be recycled after macropinocytosis of digested bone materials [120, 121]. Macropinocytosis is a non-selective endocytic pathway that consists in the uptake of nutrients and proteins from the extracellular space by invagination of the plasma membrane, which can produce changes in cell size [122]. It has been shown, that, elevated ATP levels in the extracellular space can regulate the macropinocytosis process in murine neuroblastoma cell line Neuro2a via internalization of Panx1 channel. This suggests that ATP via Panx1 and P2X7 may not only act as a chemoattractant signal in macrophages [25] and in osteoclasts [114], but as a signal to internalize Panx1 by macropinocytosis. This relationship between Panx1 and macropinocytosis occurs in the carcinogenic processes, in which ATP acts as a growth factor or energy source if internalized [123].

Furthermore, Panx1 acts as a mechanosensitive channel and as a key mediator in intercellular signaling and in inflammatory responses [110, 124]. The global Panx1 knockout mouse model has demonstrated that Panx1 is essential for load-induced skeletal responses. Both Panx1 and P2X7 receptor expression are regulated in vivo by mechanical loading in osteocyte-enriched wild-type bones in a loading time–dependent manner [125]. These findings demonstrate a functional interaction of the P2X7 receptor/Panx1 signaling complex that is crucial for osteocyte mechanosignaling. Loss of Panx1 does not affect mineral apposition rate, which represents the activity of osteoblasts, but it modulates the rate at which bone is formed, consistent with an alteration in load-induced bone remodeling [125]. Although the absence of Panx1 downregulates β-catenin expression in melanoma cells [126], β-catenin expression in non-loaded Panx1 knockout (*Panx1*^−/−^) osteocyte-enriched cells is unaffected. However, mechanical loading in *Panx1*^−/−^ bones significantly downregulates β-catenin and upregulates sclerostin, indicative of crosstalk between Panx1 and Wnt/β-catenin signaling [114]. Similarly, Seref-Ferlengez et al. have demonstrated that the complex formed by Panx1 and the P2X7 receptor and altered ATP signaling impair osteocytes and osteoblast mechanosignaling under high-glucose conditions [127].

### Panx2 in the joint

Panx2 is more ubiquitously expressed than was suggested by the initial reports that it was exclusive to the central nervous system, including cerebellum, cerebral cortex, medulla, occipital pole frontal lobe, temporal lobe, and putamen; northern blot analysis has revealed Panx2 expression in rodent eyes as well as thyroid, kidney, liver, and rat cochlear system [54, 128–130]. Using two novel monoclonal antibodies to Panx2 (N121A/1 and N121A/31), Panx2 expression has been found in murine heart, lung, stomach, spleen, spinal cord, skin, eye, colon, and testis [131]. Unfortunately, there is little data about Panx2 expression in bone or cartilage. Using predesigned TaqMan assays, Yorgan et al. studied the expression of pannexins in different tissues including spine, femur, and calvaria and found Panx2 expression by qRT-PCR in murine calvaria, but they did not observe any changes in expression during osteoblast differentiation [132].

### Panx3 in the joint

As described above, Panx3 is expressed in pre- and hypertrophic chondrocytes as well as mature osteoblasts and has been suggested to play a role in their differentiation (Fig. 2) [55, 133]. In the chondrogenic cell line ATDC5, Panx3 promotes chondrocyte differentiation by regulating intracellular ATP/cAMP levels [55]. Iwamoto et al. showed that ATP release into the extracellular space by Panx3 is responsible for PTH-mediated cell proliferation inhibition, as well as decreasing intracellular levels of cAMP and CREB phosphorylation [55]. During development, Panx3 expression at sites of intramembranous and endochondral ossification begins as early as E13–E13.5 [133]. In the growth plate, Panx3 expression precedes chondrocyte mineralization and co-localizes with bone development markers such as Col10α1 and OPN [133]. Moreover, Panx3 promoter is transactivated with runt‐related transcription factor 2 (Runx2), a key transcription factor for normal bone formation that also promotes MMP13 expression [134].

In vivo, genetic deletion of Panx3 in mice leads to abnormal differentiation of hypertrophic chondrocytes; Lac of Panx3 plays a role in joint diseases and a delay in osteoblast differentiation and mineralization [134]. Moon et al. reported elevated Panx3 abundance in damaged areas of cartilage in osteoarthritic mice and humans [9]. Global or *Cre/loxP* cartilage-selective Panx3 knockdown mice were resistant to an OA induction procedure known as destabilization of medial meniscus surgery to induce OA (DMM-OA). Furthermore, Panx3 and Panx1 levels were increased in osteophytes in DMM-operated control mice but not Panx3-deficient mice [135]. This high Panx1 and Panx3 expression results from osteophyte growth and development, that is similar to endochondral ossification and therefore leads to a similar pattern of protein expression. The altered mechanical environment that follows joint injury causes Runx2-mediated Panx3 induction, promoting ATP release from articular chondrocytes. The increase in extracellular ATP activates P2 receptors and downstream effectors such as ERK1/2. This cascade activates a vicious cycle with Runx2, Panx3,and MMP13 activation that leads to a complete destruction of the articular cartilage and joint [136–138]. It has further been reported that loss of Panx3 has no other consequence for joint development and health. This suggest that Panx1 might be sufficient to mediate normal joint development and in permanent adult cartilage [135].

In C2C12 cells, an osteoprogenitor cell line derived from mouse myoblasts, and in primary calvarial cells, Panx3 is located in plasma membrane and regulates osteoblast differentiation (Fig. 2) as its expression is induced during the transition from cell proliferation to differentiation [139]. During osteoblast differentiation, Panx3 functions as a hemichannel that releases intracellular ATP into the extracellular space. This released ATP binds to purinergic receptors in an autocrine or paracrine manner, activates the PI3K-Akt signaling pathway, and mediates the activation of endoplasmic reticulum Ca^2+^ channels due to Panx3 opening [55]. This Ca^2+^-binding activates calmodulin (CaM)/(CN) signaling pathways that, upon activation of nuclear factor of activated T-cells calcineurin-dependent1 (NFATc1), promotes the expression of Osterix and, in turn, induces the expression of osteoblast genes such as those encoding alkaline phosphatase (ALP) and osteocalcin (Ocn) [140–142]. Moreover, Panx3 activation can promote the degradation of p53, an inhibitor of osteoblast differentiation, by activating the Akt/mouse double-minute 2 homolog (MDM2) pathway. In a paracrine manner, the Panx3 gap junction propagates intracellular Ca^2+^ to neighboring cells to promote osteoblast differentiation [140, 142]. Both primary cultured osteoblasts and the MC3T3-E1 pre-osteoblast cell line express increasing levels of Panx3 as they differentiate [133]. These increases mirror those of osteogenic markers such as Alpl, Ibsp, and Sp7 during differentiation. Moreover, Panx3 expression in osteoblasts increases MDM2 phosphorylation, promotes p53 degradation, and stimulates Smad1/5 phosphorylation [55].

One mechanism specific to the function of Panx3 endoplasmic reticulum Ca^2+^ channels is the phosphorylation of serine 68 (Ser68) of Panx3 to promote osteoblast differentiation [143]. Mutation of this residue to alanine was sufficient to inhibit Panx3-mediated osteoblast differentiation as it induces depletion of Osterix and ALP expression. Panx3 Ser68 phosphorylation has been found in pre-hypertrophic and hypertrophic chondrocytes, bone areas of the newborn growth plate, and the endoplasmic reticulum membranes in C2C12 cells [143]. These data indicate that this phosphorylation is an essential step controlling the gating of the Panx3 endoplasmic reticulum Ca^2+^ channel to promote osteogenesis, and that it is necessary for osteoblast differentiation but not osteoprogenitor proliferation. Several mechanisms have been proposed whereby this Ser68 phosphorylation of Panx3 at the endoplasmic reticulum membrane might be modulated by a cytoplasmic kinase. It is possible that a protein complex formed by Akt and other molecules, and a signaling pathway they control, may be necessary to activate Panx3 as a Ca^2+^ channel [143]. 5′ AMP-activated protein kinase (AMPK) can form a complex with Akt to activate Panx3 endoplasmic reticulum Ca^2+^ channel. AMPK activity regulates Panx3 proliferation and the differentiation of odontoblasts through ATP-releasing hemichannel activities [144]. BMP2 is another candidate for forming a complex with Akt, because it regulates Panx3 expression and the activation of the Panx3 signaling pathway [140, 141]. Both Smad-dependent signaling and an independent pathway, such as MAPK signaling, may interact to produce Panx3 endoplasmic reticulum Ca^2+^ channel activation. Another residue important for Panx3 activity (though less so than Ser68) is Ser303, whose mutation to alanine inhibits Panx3-mediated ALP activity [143]. All together, these results suggest that Panx3 phosphorylation occurs at an early stage and declines during development. This may be because in the transition from proliferation to differentiation, cells normally require substantial energy flow, with enormous exchanges of energy, and this might be regulated by phosphorylation of Panx3 in the endoplasmic reticulum to control intracellular Ca^2+^ levels.

Panx3 knockdown in zebrafish causes delayed osteoblast differentiation and mineralization and cartilage deformity [134]. Similarly, osteoblast differentiation is induced by Panx3-mediated ATP release and downstream PI3K/AKT signaling, and KO zebrafish demonstrated delayed osteoblast differentiation and mineralization [7, 10]. Investigations of animals lacking Panx3 either in the osteoblast lineage or ubiquitously indicate that Panx3 expression in osteoblasts is not required for postnatal bone remodeling. As described previously for cartilage, it is possible that the loss of Panx3 in both *Panx3* conditional knockout (*Panx3*^*fl/fl*^; *Runx2-Cre*) and *Panx3*^−/−^ mice vs. their phenotypically normal littermates can be compensated by the action of Panx1 [132].

## Conclusions and future directions

In this review, we have discussed the presence of connexins and pannexins in joint tissue and their relevance to the initiation of rheumatic diseases such as OA and RA. Chondrocytes, osteoblasts, and osteoclasts, the main cell types found in joints, all express one or more isoforms of both connexins and pannexins. Moreover, the regulation of ATP channels, including connexins and pannexins, is altered as a consequence of joint diseases.

Among the connexins, Cx43 levels in cartilage and synovial membrane are elevated during the onset of disease and inflammation [63, 78–80]. Bone is also affected, as Cx43 prevents early osteoblast differentiation [89, 90]. There is disagreement about whether the age-associated reduction of Cx43 in osteoblasts might contribute to bone loss and osteoporosis [92]. However, studies to date seem to indicate that inhibiting the reduction of Cx43 in joint tissues might be beneficial in preventing the progression of joint diseases, taking into account the inflammatory component of the disease pathologies as well as the relevant role of MMPs in these pathologies [64, 81].

Among the pannexins, Panx1 mediates the response to stiffness in cartilage but also joint pain [63, 102, 103]. As pain is the main factor in joint disease causing disability, its attenuation is a very attractive target when developing treatments, especially considering that previous strategies targeting pain mediators have been unsuccessful [145].

In addition, Panx1/P2X7 receptor complex function and ATP release together act as a ‘find me’ signal necessary for macrophage recruitment [42], osteocyte apoptosis, and enhanced bone resorption [113, 117]. This could have implications for treating bone pathologies such as osteoporosis, and preliminary studies have begun to harness its potential as a means to prevent bone loss [146].

Panx3 is involved in the cartilage damage seen in OA in both mice and humans [9] and also promotes hypertrophic chondrocyte differentiation [55, 133]. This is significant especially when considering that chondrocytes from OA patients show a hypertrophic-like phenotype, and preventing the development of this phenotype has been proposed to slow or prevent OA progression [147].

To further clarify this, however, we will need to better understand whether hemichannels exist in cells of the osteoblastic lineage and whether their controversial roles described herein represent real effects. Also, further investigation will be needed to elucidate the different roles of gap junctions as compared to hemichannels in joint tissues and how their interplay might affect joint disease.

In conclusion, although we focus here on OA and RA, improved understanding the modulation of connexins and pannexins in joint tissues could provide attractive treatment approaches that could also benefit the treatment of all joint diseases s, which are increasingly needed given that the aging of the population, along with the rising prevalence of metabolic dysfunction, are increasing the socioeconomic burden of joint diseases with every year that passes.

## Data Availability

Data sharing not applicable—no new data generated.
